# Acute Inflammatory Responses of Nanoparticles in an Intra-Tracheal Instillation Rat Model

**DOI:** 10.1371/journal.pone.0118778

**Published:** 2015-03-04

**Authors:** Andrea L. Armstead, Valerie C. Minarchick, Dale W. Porter, Timothy R. Nurkiewicz, Bingyun Li

**Affiliations:** 1 Biomaterials, Bioengineering & Nanotechnology Laboratory, Department of Orthopaedics, School of Medicine, West Virginia University, Morgantown, West Virginia, United States of America; 2 Pharmaceutical and Pharmacological Sciences Graduate Program, School of Pharmacy, West Virginia University, Morgantown, West Virginia, United States of America; 3 Department of Physiology and Pharmacology, School of Medicine, West Virginia University, Morgantown, West Virginia, United States of America; 4 Center for Cardiovascular and Respiratory Sciences, Robert C. Byrd Health Sciences Center, School of Medicine, West Virginia University, Morgantown, West Virginia, United States of America; 5 Pathology and Physiology Research Branch, Health Effects Laboratory Division, National Institute for Occupational Safety and Health, Morgantown, West Virginia, United States of America; 6 Mary Babb Randolph Cancer Center, Morgantown, West Virginia, United States of America; Chinese Academy of Sciences, CHINA

## Abstract

Exposure to hard metal tungsten carbide cobalt (WC-Co) “dusts” in enclosed industrial environments is known to contribute to the development of hard metal lung disease and an increased risk for lung cancer. Currently, the influence of local and systemic inflammation on disease progression following WC-Co exposure remains unclear. To better understand the relationship between WC-Co nanoparticle (NP) exposure and its resultant effects, the acute local pulmonary and systemic inflammatory responses caused by WC-Co NPs were explored using an intra-tracheal instillation (IT) model and compared to those of CeO_2_ (another occupational hazard) NP exposure. Sprague-Dawley rats were given an IT dose (0-500 μg per rat) of WC-Co or CeO_2_ NPs. Following 24-hr exposure, broncho-alveolar lavage fluid and whole blood were collected and analyzed. A consistent lack of acute local pulmonary inflammation was observed in terms of the broncho-alveolar lavage fluid parameters examined (i.e. LDH, albumin, and macrophage activation) in animals exposed to WC-Co NP; however, significant acute pulmonary inflammation was observed in the CeO_2_ NP group. The lack of acute inflammation following WC-Co NP exposure contrasts with earlier *in vivo* reports regarding WC-Co toxicity in rats, illuminating the critical role of NP dose and exposure time and bringing into question the potential role of impurities in particle samples. Further, we demonstrated that WC-Co NP exposure does not induce acute systemic effects since no significant increase in circulating inflammatory cytokines were observed. Taken together, the results of this *in vivo* study illustrate the distinct differences in acute local pulmonary and systemic inflammatory responses to NPs composed of WC-Co and CeO_2_; therefore, it is important that the outcomes of pulmonary exposure to one type of NPs may not be implicitly extrapolated to other types of NPs.

## Introduction

The increased use of engineered nanomaterials (ENMs) in commercial manufacturing and consumer products presents an important toxicological concern. As the ENMs are used repetitively and wear over time, nanoparticles (NPs) are generated and released into the environment, thereby creating a NP exposure hazard. Currently, there are no definitive “standards” for evaluating the toxic effects of NPs, so identifying NP exposure effects remain a challenge for researchers world-wide [1]. It is evident from the literature that the effects of NP exposure effect vary greatly, ranging from non-toxic to carcinogenic, depending upon the particle size, composition, dose, length, and route of exposure [1–6]. The pulmonary effects of NPs are particularly important, as airborne NPs are inhaled and inhalation is the most frequent route by which workers are exposed in occupational settings [7–9].

Recently, it has been reported that inhaled NPs are capable of depositing in the lung and causing systemic effects at sites distant from that of exposure [6, 10, 11]. Translocation of NPs across the lung and into the bloodstream may result in NP deposition in other organs (liver, spleen, kidney), with subsequent organ damage or toxicity, and may cause changes in vascular function or permeability [6, 10, 12–19]. It is difficult to predict the long-term impact of these systemic effects, so the extent by which systemic effects of NP exposure may contribute to or alter specific disease states remains unknown.

As mentioned above, occupational inhalation of NPs is of particular concern; specifically, exposure to tungsten carbide cobalt (WC-Co) dusts and particles. WC-Co is a hard composite metal commonly used as a material and coating for equipment used in mining and drilling industries [20]. As these tools are used extensively in a closed environment, WC-Co dusts containing particles of respirable range are released, thereby creating an occupational inhalation hazard [21, 22]. Inhalation of WC-Co containing dusts and particles is known to cause hard metal lung disease (HMLD) and a two-fold increased risk for lung cancer [23–27]; however, the relationship between acute WC-Co toxicity and the potential role of inflammation on HMLD progression remains unknown. The toxicity of WC-Co particles toward a number of cell types *in vitro* has been reported in the literature [28–42]. Specifically, we recently found that WC-Co particles in the nano-size range were internalized by epithelial cells and that exposure to WC-Co NPs resulted in significant toxicity toward lung epithelial cells at concentrations as low as 10 μg/mL for exposure periods as short as 0.5 hr, significant toxicity at concentrations of 0.1 and 1 μg/mL after 48 hr exposure and that, overall, WC-Co NPs caused significantly greater toxicity compared to WC-Co micro-particles [42].

Additionally, there have been several studies regarding the toxicity of WC-Co particles *in vivo* [43–50]. These early *in vivo* studies focused on the local pulmonary responses to WC-Co exposure and confirmed that the composite material of WC-Co was responsible for the observed toxic effects when compared to tungsten (W), carbide (C), or cobalt (Co) exposure alone [43, 46, 47]. The WC-Co particles used for these studies were within the 2–4 μm size range and reported toxicity following single intra-tracheal instillation (IT) exposure was marked by severe alveolitis, pulmonary edema, and increased levels of lactate dehydrogenase (LDH), which were observed after 24 hr and up to 72 hr post-exposure [47, 48, 50]. While the findings regarding the pulmonary toxicity of WC-Co micro-particles were fairly consistent among these studies, there is a lack of information regarding the toxicity of WC-Co particles in the nano-size range *in vivo*.

Given the gap in knowledge regarding nano-sized WC-Co toxicity *in vivo* and our recent findings demonstrating the enhanced toxicity of nano-sized WC-Co compared to micro-sized WC-Co *in vitro*, we conducted a pilot study to examine the acute pulmonary and systemic inflammatory effects of WC-Co NP exposure, which have not yet been reported, using an intra-tracheal instillation rat model and compared the outcomes with cerium dioxide (CeO_2_) NPs. The Nurkiewicz laboratory, including Minarchick, Porter, and Nurkiewicz whom are coauthors of this study, previously reported that CeO_2_ NPs induced microvascular dysfunction following pulmonary exposure *in vivo*, characterized by impaired endothelium-dependent and endothelium-independent dilation and speculated that such microvascular changes may likely contribute to cardiovascular dysfunction associated with particle exposure [16]. In this case, we hypothesized that WC-Co NPs would induce dose-dependent acute pulmonary inflammation, similar to CeO_2_ NPs [16, 51, 52] and may cause systemic inflammation marked by increased levels of inflammatory cytokines such as tumor necrosis factor alpha (TNF-α) and interleukin 6 (IL-6).

## Materials and Methods

### WC-Co and CeO_2_ NPs

Tungsten carbide cobalt (WC-Co) NPs were purchased from Inframat Advanced Materials (Manchester, CT). Cerium dioxide (CeO_2_) NPs were synthesized and characterized as previously described [16]. Stock solutions of WC-Co and CeO_2_ NPs were prepared as previously reported [16]. Briefly, dry WC-Co or CeO_2_ NPs were weighed and added to 10 mL of saline (Normosol) with 10% fetal bovine serum (FBS) and sonicated over ice to ensure dispersion. Previous studies showed that saline and FBS reduced particle aggregation and did not induce mechanical artifacts in terms of broncho-alveolar lavage (BAL) and systemic responses in rats [16, 53, 54]. The average size of WC-Co and CeO_2_ NPs in Normosol (isotonic saline) plus 10% FBS was determined via dynamic light scattering (DLS) using a Malvern Zetasizer version 7.01 (Malvern Instruments Ltd., Malvern, UK). WC-Co NP were also characterized using transmission electron microscopy (TEM) and scanning electron microscopy (SEM) for confirmation of size and electron-dispersive x-ray (EDX) to determine elemental composition. For morphological examination via TEM, WC-Co particles were diluted in distilled water and vortexed for 60 sec to remove traces of salt and protein from the original suspension which could interfere with TEM imaging. Five microliters of the resulting suspension were then transferred to a carbon-coated copper grid and allowed to dry at room temperature before imaging on a Zeiss Libra 120 electron microscope at 120 kV (Carl Zeiss Microscopy, Jena, Germany). The elemental composition of WC-Co NP was determined via SEM/EDX on a JEOL JSM 7600F equipped with an Oxford Instruments energy dispersive x-ray (EDX) system. EDX measurements were carried out in the Point & ID mode with spectrum acquisition time of 120 s and spectrum range of 0–10 keV.

### Animals

Male Sprague-Dawley rats (8–9 weeks old) were purchased from Hilltop Laboratories (Scottdale, PA). The rats were housed at the West Virginia University animal facility in ventilated cages, under controlled humidity and temperature, with a 12 hr light/dark cycle with food and water provided *ad libitum*. Animals were acclimated for at least 2 days prior to use. Rats were divided randomly into groups (six animals per group) and assigned to either the 0, 50, 250, or 500 μg WC-Co or 400 μg CeO_2_ NP group. All procedures were approved by the West Virginia University Animal Care and Use Committee (Protocol Number 12–0414) and carried out in accordance with recommendations set forth in the Guide for the Care and Use of Laboratory Animals by the National Institutes of Health. All efforts were made to ensure minimal suffering during stated procedures.

### Intra-Tracheal Instillation Rat Model and NP Exposure

The NP stock solutions were sonicated for 5 min on ice to ensure particle dispersion and used immediately for IT instillation. Rats were lightly sedated with isofluorane gas (5% induction) and intra-tracheally instilled with a 300 μL bolus dose of the stock NP solutions to achieve final doses of 0, 50, 250, and 500 μg WC-Co NPs or 400 μg CeO_2_ NPs. Rats were monitored after instillation until consciousness was regained. After a 24-hr recovery period, rats were anesthetized with thiobutabarbital sodium salt hydrate (Inactin; Sigma-Aldrich, MO) at a dose of 1 mg/kg via intra-peritoneal (i.p.) injection. Anesthesia was confirmed by testing the toe-pinch reflex. Upon euthanization, the rat abdomen was opened and whole blood was collected in anti-coagulant (ethylenediaminetetraacetic acid, EDTA) vacuum tubes via the abdominal aorta until a minimum of 6 mL blood was obtained. Following blood collection, the aorta was cut for complete exsanguination and broncho-alveolar lavage (BAL) was performed immediately thereafter.

### Blood Plasma Isolation

Whole blood samples were kept on ice until all samples were collected; samples were then centrifuged at 2000 × g for 15 min to separate the plasma from the cellular blood components. The plasma (supernatant) was drawn off using a pipet, transferred to a cryogenic vial in 0.5 mL aliquots, and flash frozen in liquid nitrogen for later cytokine analysis.

### Assessment of Pulmonary Inflammation at 24-hr Post-Exposure

Pulmonary inflammation was assessed in the BAL fluid after NP exposure by evaluating several parameters. First, BAL fluid samples were assessed for cytotoxicity using the LDH assay and second, albumin protein concentration in the BAL fluid was determined to evaluate the integrity of the epithelial-endothelial (blood-gas exchange) barrier in the lung. Third, inflammatory cells were isolated from the BAL fluid and differential cell counts performed to identify the number of alveolar macrophages (AM) and polymorphonuclear leukocytes (PMN) present in the lung following NP exposure. Further, isolated AM activation states were examined using a standard chemiluminescence assay. Then, the concentration of inflammatory cytokines (i.e. TNF-α, IL-6, and IFN-γ) were determined in BAL fluid samples using enzyme-linked immunosorbent assay (ELISA).

**BAL Procedure and BAL Fluid Collection.** Broncho-alveolar lavage (BAL) was performed with Ca^2+^/Mg^2+^-free phosphate buffered saline (PBS, pH 7.4) plus 5.5 mM D-glucose as previously described [18]. Briefly, a tracheal cannula was inserted and BAL was performed through the cannula using ice-cold PBS. The first BAL fluid, totaling 6 mL of PBS, was collected and immediately centrifuged (650 x g, 10 min, 4°C). The resulting first BAL fluid supernatant was then divided for later analysis: two 0.5 mL aliquots were flash-frozen in liquid nitrogen for cytokine determination by ELISA and the remaining 5 mL was kept on ice for analysis of LDH and albumin. After the first BAL was collected, BAL was repeated using 8 mL of PBS until a total of 40 mL BAL fluid was collected. Next, the 40 mL of BAL fluid was centrifuged (650 x g, 10 min, 4°C) and the resulting cell pellet was pooled with the cell pellet from the first BAL fluid. The pooled cells were re-suspended in HEPES-buffered medium (10 mM N-[2-hydroxyethyl]piperazine-N′-[2-ethanesulfonic acid], 145 mM NaCl, 5 mM KCl, 1 mM CaCl_2_, and 5.5 mM D-glucose, pH 7.4) and centrifuged a second time (650 x g, 10 min, 4°C). The resulting supernatant was decanted and a final suspension of the isolated BAL cells was prepared in HEPES-buffered medium.

**Albumin Protein Assay and LDH Activity.** LDH and albumin assays were performed as previously described [16, 55] on the same day as BAL fluid collection using a Roche Cobas c111 (Roche Diagnostic Systems, Indianapolis, IN). In brief, LDH activity was used as a marker of cytotoxicity. A commercial assay kit was purchased from Roche Diagnostic Systems and used to measure LDH activity based on the LDH-driven oxidation of pyruvate coupled with the reduction of nictoinamide adenine dinucleotide at 340 nm. Albumin concentration was monitored as an indicator of cellular integrity using a commercially available kit from Sigma Chemical Co. (St. Louis, MO) based on albumin binding to bromcresol green and measuring the color change at 628 nm.

**Histology.** A total of 1.0 × 10^6^ BAL cells were suspended in 200 μL HEPES-buffered medium and transferred to microscope slides using the cytospin approach [56]. The resulting cytospin preparations were stained with modified Wright-Giemsa stain and examined for the presence of WC-Co particles (black dots in appearance). Cell differentials were determined using light microscopy. Differential cell counts were calculated by multiplying the total cell count by the cell differential percentage obtained from the cytospin preparations.

**Macrophage Chemiluminescence.** The activation state of alveolar macrophages (AM), previously isolated from the BAL fluid (above), was determined in a total volume of 0.5 mL HEPES-buffered medium as previously described using a chemiluminescence assay [57]. First, chemiluminescence of resting AM (non-stimulated) was determined by incubating 1.0 × 10^6^ AM/mL at 37°C for 20 min, followed by the addition of 5-amino-2,3-dihydro-1,4-phthalazinedione (luminol) to a final concentration of 0.08 μg/mL. The resulting chemiluminescence was measured with an automated luminometer (Berthold Autolumat Plus LB 953, Oakridge, TN) at 390–620 nm for 15 min, where the integral of counts per minute (cpm) versus time was calculated. Next, zymosan-stimulated chemiluminescence was determined by adding 2 mg/mL of un-opsonized zymosan just prior to the measurement of chemiluminescence. The use of un-opsonized zymosan in this assay allows for the determination of AM chemiluminescence, which is a reflection of the macrophage activation state, because un-opsonized zymosan stimulates AM chemiluminescence [58] but does not stimulate polymorphonuclear leukocyte (PMN) chemiluminescence [59, 60]. Stimulated macrophage chemiluminescence was then calculated by subtracting the cpm from the resting AM measurement from the cpm of the zymosan-stimulated measurement.

### Inflammatory Cytokine ELISA

Standard curves for cytokines including TNF-α, IL-6, and Interferon (IFN-γ) were prepared using a dilution series with a commercial ELISA kit (Signosis, Inc., Santa Clara, CA). Previously frozen plasma and BAL fluid samples were thawed and used to determine the cytokine concentrations in each sample. Briefly, 100 μL of BAL fluid or plasma sample was added to each well of the 96-well ELISA plate and incubated for 2 hr to allow sufficient binding to the immobilized antibodies within each well. Samples were then aspirated and wells were rinsed three times with 200 μL buffer per wash. Next, 100 μL biotin-labeled detection antibody was added to each well and incubated for 1 hr. The washing step was repeated followed by the addition of 100 μL streptavidin-horseradish peroxidase (HRP) conjugate to each well. After 45 min, the washing step was repeated and 100 μL substrate was added to each well. The plate was further incubated for 30 min in the dark, followed by the addition of 50 μL stop solution to each well. The absorbance of each sample was immediately measured at 450 nm. BAL fluid and plasma samples were run in triplicate and the cytokine concentration of each sample was calculated based on the sample absorbance and the slope of the standard curve for each respective cytokine.

### Statistical Analysis

All data were presented as the mean ± standard deviation. Statistical significance between experimental groups was determined using one-way analysis of variance (ANOVA) and Dunnett’s post-hoc analysis in GraphPad Prism 6 software (San Diego, CA).

## Results

### WC-Co NP Characterization

**Fig 1:**
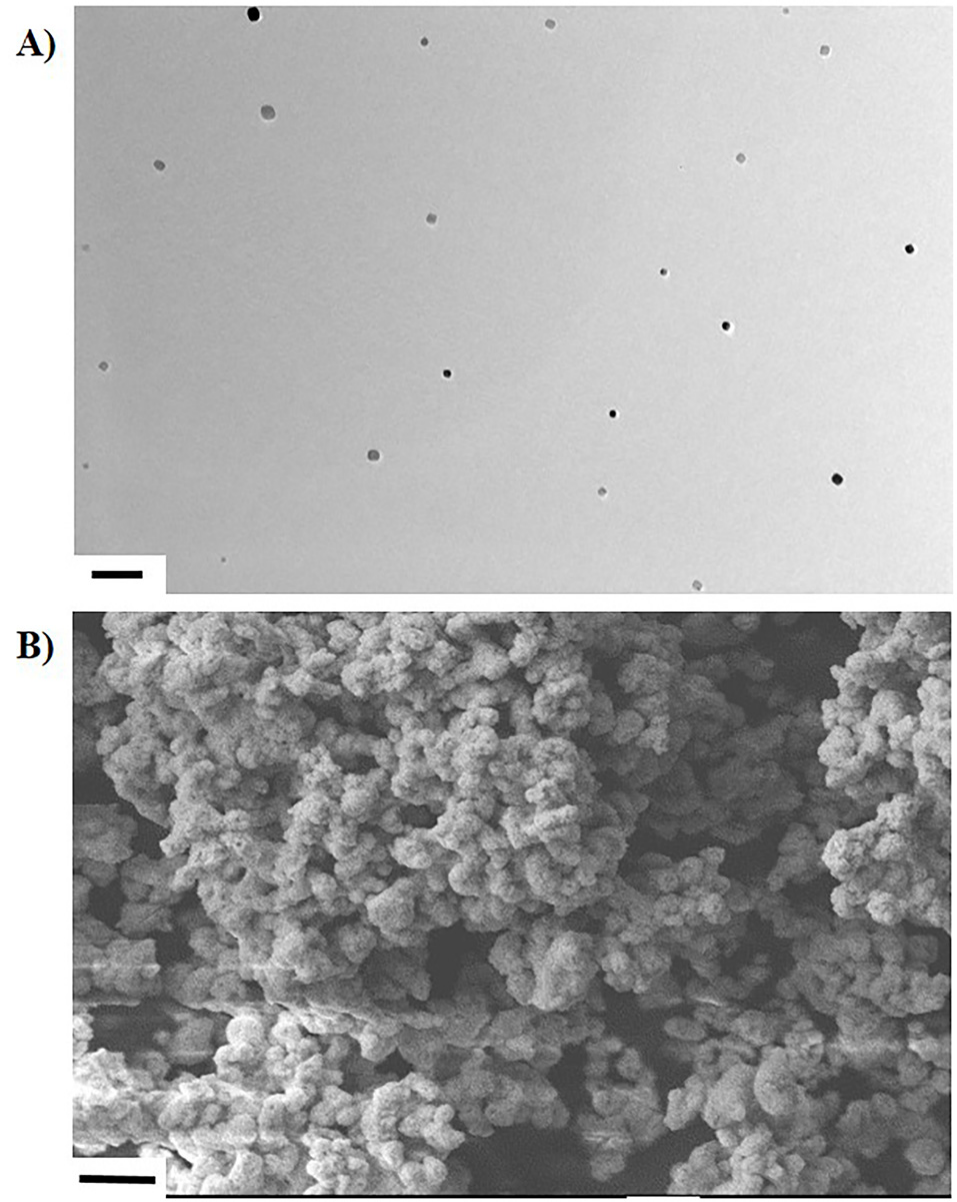
Characterization of WC-Co NP via A) TEM (scale bar = 500 nm) and B) SEM (scale bar = 1 μm).

The average size of WC-Co and CeO_2_ NPs were determined to be approximately 100 nm and 190 nm, respectively, as prepared in suspension for IT (Normosol containing 10% FBS). This finding correlated with our TEM and SEM imaging of WC-Co NPs, which qualitatively indicated that WC-Co NPs were approximately 100 nm in diameter (Fig. 1). As shown in Table 1, the chemical composition of WC-Co NPs included 72.1 wt.% W, 13.4 wt.% Co, 7.6 wt.% C, and 6.8 wt.% O as reported previously [42].

**Table 1:** Summary of the characteristics of WC-Co NP, including size and elemental composition.

Average Size			Elemental Composition			
DLS	TEM	SEM				
98 nm	~ 100 nm	~ 100 nm	72.1% W	13.4% Co	7.6% C	6.4% O

### Pulmonary Inflammation

**Fig 2:**
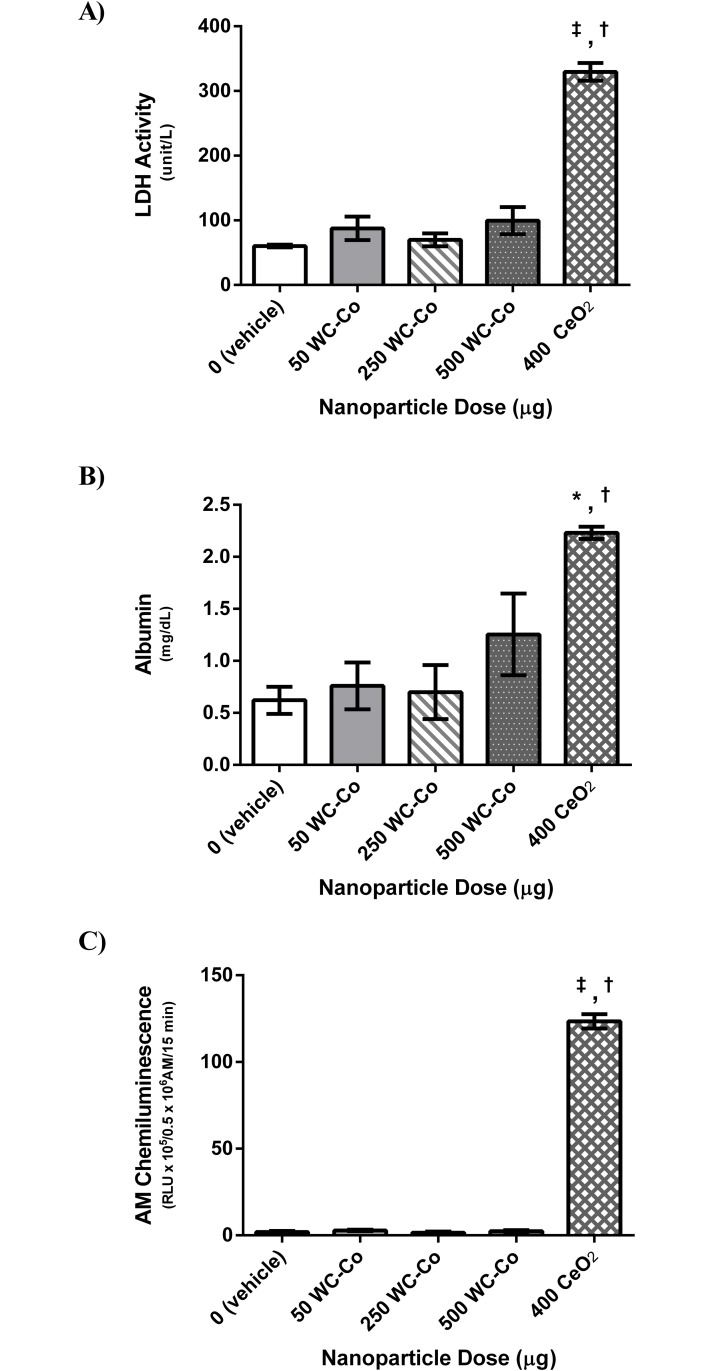
Pulmonary inflammation parameters assessed in the BAL fluid following 24-hr exposure to WC-Co and CeO_2_ NPs: A) LDH activity, B) albumin, and C) AM chemiluminescence. Values presented as mean ± SD. (*P < 0.05, ‡P < 0.001 compared to the vehicle control, and †P < 0.01 compared to WC-Co NP exposed groups)

BAL fluid was collected and analyzed to assess pulmonary inflammation following 24-hr exposure to WC-Co or CeO_2_ NPs. Compared to the vehicle control group, there were no significant differences in LDH activity for WC-Co NP exposed animals at the doses studied. A significant increase in LDH activity was observed in the CeO_2_ NP group compared to the vehicle control and all of the WC-Co NP exposed groups (Fig. 2A). This indicated a lack of cytotoxicity in the WC-Co NP exposed groups at the doses studied while the exposure to CeO_2_ NPs caused significant cytotoxicity. Similarly, there were no significant differences found in the albumin content in WC-Co NP exposed animals compared to vehicle control, although relatively higher albumin content was observed at the exposure dose of 500 μg compared to the other doses (i.e. 50 and 250 μg) (Fig. 2B). A significant increase in albumin was found in the CeO_2_ NP exposed group compared to the vehicle control and all of the WC-Co NP exposed groups (Fig. 2B). This indicated that the epithelial-endothelial barrier remained undisrupted in WC-Co NP exposed animals but was affected in the CeO_2_ NP exposed group.

**Fig 3:**
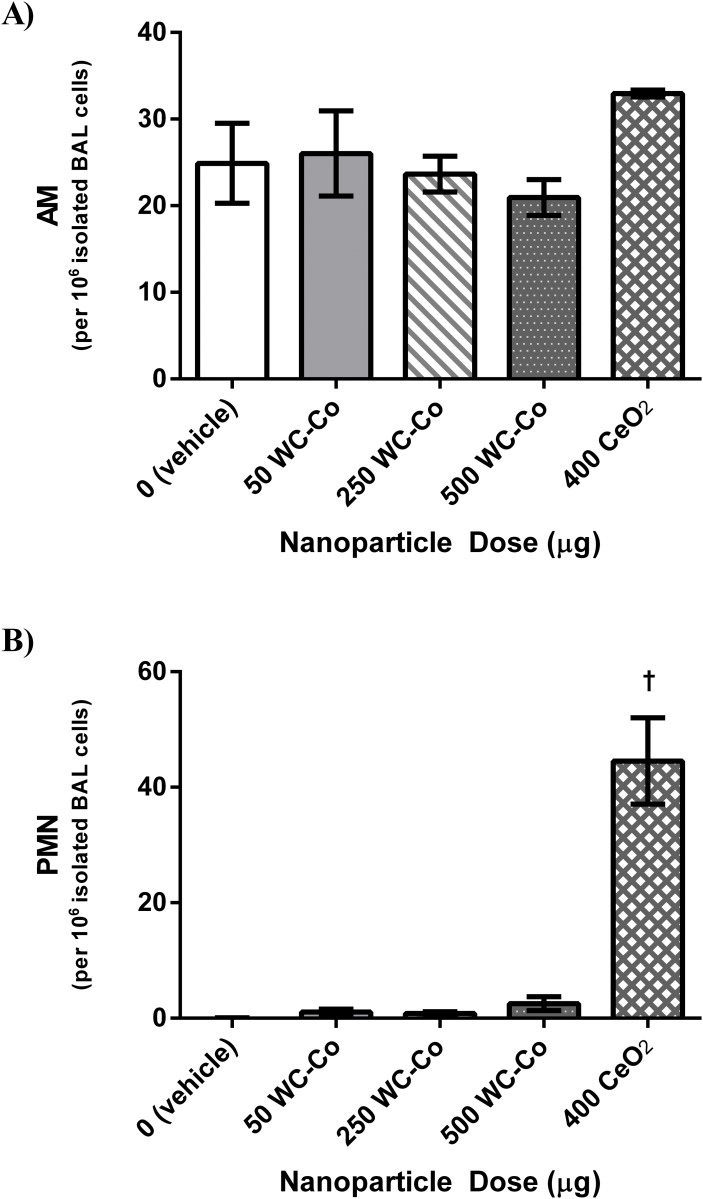
Inflammatory cells quantified in BAL fluid samples following 24-hr exposure to WC-Co and CeO_2_ NPs: A) alveolar macrophages (AM) and B) polymorphonuclear leukocytes (PMN), represented as the total number of AM/PMN per 10^6^ isolated BAL cells per rat. Values presented as mean ± SD. (†P < 0.01 compared to the vehicle control and WC-Co NP exposed groups)

The activation state of AM was determined via zymogen-stimulated chemiluminescence assay, where no significant differences were found in AM activation in WC-Co NP exposed animals at all the doses studied compared to the vehicle control group. A significant increase in AM activation was observed when the CeO_2_ NP exposed group was compared to the vehicle control and to all of the WC-Co NP exposed groups (Fig. 2C). Moreover, the number of AMs in the BAL fluid samples was similar across the vehicle control and all WC-Co NP exposed animals, where a relatively higher number of AMs was found in the CeO_2_ NP exposed group compared to the vehicle control and WC-Co NP exposed groups; however, the differences were not significant (Fig. 3A). Additionally, no significant differences in the number of PMNs were found between the WC-Co NP exposed groups and the vehicle control group; however, a significant increase in the number of PMNs was detected in the CeO_2_ NP exposed group compared to the vehicle control and the WC-Co NP exposed groups (Fig. 3B).

**Fig 4:**
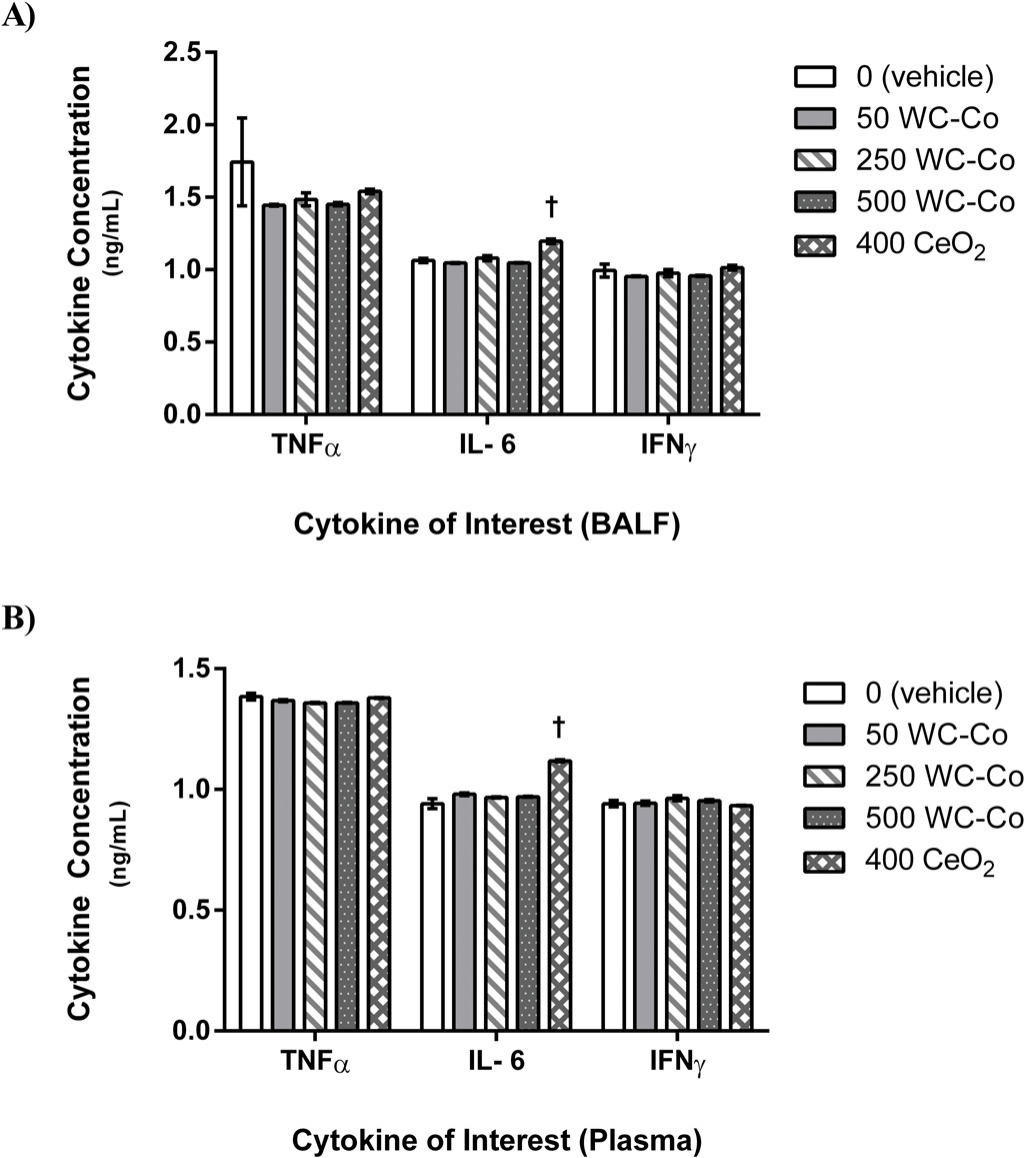
Inflammatory cytokine concentrations in A) BAL fluid and B) blood plasma. (†P < 0.05 compared to the vehicle control and WC-Co NP exposed groups)

Further, no significant differences were detected in the levels of inflammatory cytokines (i.e. TNF-α and IFN-γ) in BAL fluid among the WC-Co, CeO_2_, and vehicle control groups (Fig. 4A) with the exception of IL-6, where a significant (P = 0.049) increase in IL-6 was observed for the CeO_2_ NP group compared to the vehicle control and WC-Co NP exposed groups (Fig. 4A).

### Systemic Inflammation

The levels of inflammatory cytokines including TNF-α, IL-6, and IFN-γ were determined in blood plasma samples to examine the potential systemic inflammatory response to WC-Co and CeO_2_ NP exposures. No significant differences were observed in TNF-α and IFN-γ levels among all the animal groups studied (i.e. Control, WC-Co NP, and CeO_2_ NP groups), but significantly (P = 0.049) higher IL-6 was found in the CeO_2_ NP exposed group compared to the vehicle control and WC-Co NP exposed groups (Fig. 4B).

### Isolated BAL Cell Histology

**Fig 5:**
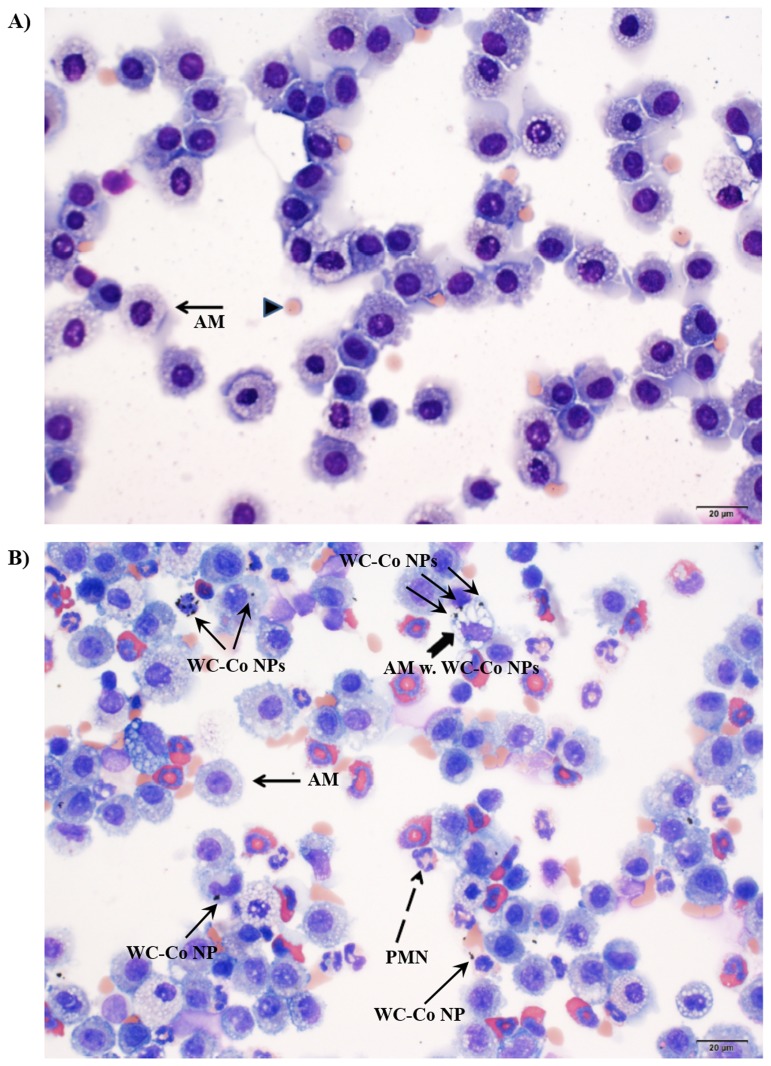
Histology of isolated BAL fluid cells from a representative A) control (vehicle only) rat and B) 500 μg WC-Co NP exposed rat. Scale bars = 20 μm. (black arrow = alveolar macrophage, AM; arrow head = erythrocyte; dotted arrow = polymorphonuclear leukocyte, PMN; wide arrow = AM with WC-Co NPs)

Histological examination of the cytospin cell preparations revealed a population of AMs present in both the vehicle control (Fig. 5A) and WC-Co NP exposed groups (Fig. 5B). AM containing NPs were visible in WC-Co exposed groups (Fig. 5B), where WC-Co NPs were visible as distinct black dots within the AM (denoted by arrows in Fig. 5B), which were not observed in the control (vehicle only) group (Fig. 5A). These data suggest that AM were capable of phagocytizing the WC-Co NP; however, the overall lack of inflammation observed in the other pulmonary parameters suggests that the WC-Co NPs were recognized as ‘inert’ by the AM and did not cause significant AM activation.

## Discussion

In this study, we determined the acute inflammatory effects of WC-Co and CeO_2_ NP exposure in terms of local pulmonary responses via assessment of BAL fluid and the acute systemic effects via quantification of important inflammatory mediators in the blood.

In general, the presence of particles in the lungs, including NPs, is thought to promote the recruitment of macrophages, increase macrophage phagocytic activity and thereby stimulate particle clearance from the lung [61–64] as part of the normal physiological response. Macrophage recruitment and phagocytosis of deposited particles is rapid, usually occurring within 24 hr of exposure for most animal species [62]. In this study, WC-Co NPs were phagocytized by AMs after 24 hr, evidenced by histological examination, which is consistent with reports demonstrating the uptake of other NPs such as graphene [63], titanium dioxide [65, 66], and magnetite [67] by AMs *in vivo*. Interestingly, WC-Co NPs were also “phagocytized” by lung bronchial epithelial cells *in vitro* [42], which suggests that NP internalization may not be exclusive to macrophages and is of particular interest, since hard metal (WC-Co) deposits have been found in workers diagnosed with HMLD [68–70]. In the present study, WC-Co NPs did not induce significant acute pulmonary inflammation, compared to the vehicle control, in the assessment of LDH activity and albumin content in the BAL fluid following 24-hr exposure at doses of 50–500 μg per rat. The lack of acute pulmonary inflammation is further supported by the observation that WC-Co NP exposure caused little change in the number of AM and PMN cells and did not increase macrophage activation following 24-hr WC-Co NP exposure. This outcome is similar to that reported for instilled titanium dioxide NPs, which do not cause any substantial acute pulmonary inflammation after 24 hr at a dose up to 200 μg per rat [65, 66].

It is known that particle size may play a major role in the depth of tissue penetration and toxicity. Compared to micron-sized particles, NPs are smaller and have higher surface area (that is available for tissue interaction) and are thereby capable of deeper penetration and possessing higher toxicity [7, 71–76]. However, no significant alterations in LDH activity and albumin levels were observed in this study following WC-Co NP exposure, while significant increases in LDH and albumin were reported in a similar IT rat model following 24-hr exposure of WC-Co in the 2 μm size range [44, 48, 50]. Two factors may have contributed to the differences observed between this study and the previous ones: particle dose and chemical composition.

Currently, there are no occupational exposure limits defined for WC-Co, so it is difficult to define appropriate dosing schemes for *in vivo* examination. However, because WC-Co NP exposure primarily occurs in industrial environments (such as hard metal manufacturing or mining/drilling), inhalation of WC-Co NPs over time will result in NP accumulation in the lung which could be substantial, depending on how long the person works in such an environment. In this study, we wanted to define an appropriate WC-Co NP dosage for acute exposure, which would be representative of a cumulative WC-Co NP inhalation in an industrial environment. We considered the results from our previous *in vitro* toxicity studies in lung epithelial cells, where we reported significant WC-Co NP toxicity *in vitro* at dosages ranging from 10–1000 μg/mL [42] and on the previous *in vivo* findings regarding pulmonary CeO_2_ NP toxicity in the microgram range. Microgram doses of CeO_2_ NP are known to cause significant inflammation in exposed animals [16] and this dosage is considered occupationally relevant, as the microgram dose approximates the total lung deposition of particulate matter in a person working roughly 30 years in an industrial environment [16]. Given this information and the overall lack of exposure limits for WC-Co NP, for this study, we elected to dose our animals based on total lung burden in the microgram range (50–500 μg per rat), which lies within the toxic range of WC-Co NP *in vitro* against lung epithelial cells.

Our selection of a total microgram NP dosage contrasts with the dosages in previous WC-Co NP studies [16, 44, 48, 50], which used a mg per kg body weight dosing scheme with total WC-Co NP doses ranging from 3–30 mg per rat in a single IT exposure. It is possible that these previous studies may have overloaded the lung [77–80] and caused significant inflammation based on particle load rather than the presence of WC-Co itself. It has been suggested that “particle overload” in rat lungs can occur at particle concentrations of 1 mg per g of lung weight [77]; given the average lung weight of 1.5–1.9 g per rat, particle overload could occur at total pulmonary particle dosages of 2 mg or higher [77, 79] regardless of the material. Previously, significant pulmonary inflammation was reported in rats exposed to 16.67 mg micro-sized WC-Co per kg body weight [16, 44, 48, 50], a total lung burden of WC-Co particles which lies within the range of particle overload dose according to the literature. We believe that the microgram dosage of WC-Co NP applied in our study is well under the overload dose; therefore, the lack of inflammatory response observed may be due to this phenomenon.

Additionally, in the previous studies, WC-Co particles were obtained from hard metal manufacturing facilities and were reported to contain a significant amount of iron [44, 48, 50], which is not found in our WC-Co NPs (see Table 1). Iron has recently been identified as a pulmonary irritant [81–83] and could have contributed to the observed inflammatory responses in the previous studies. In our case, elemental analysis of our WC-Co NP indicated that no contaminants were present and we can therefore attribute the low level of inflammation caused by acute pulmonary exposure WC-Co NPs to WC-Co itself.

In this study, no significant differences in inflammatory cytokines (i.e. TNF-α, IL-6, IFN-γ) were found in plasma or BAL fluid samples for WC-Co NP exposed animals compared to the vehicle control. These findings indicate that WC-Co NPs did not induce acute systemic inflammation after 24-hr pulmonary exposure at the doses studied. By contrast, within the emerging body of literature regarding NP toxicity *in vivo*, it is reported that cadmium oxide [84], titanium dioxide [85], and silver [86] NPs are capable of inducing systemic inflammation after acute pulmonary exposure, marked by increased inflammatory cytokine levels. For example, a three-fold increase in the pulmonary levels of TNF-α and IFN-γ were observed in mice exposed to cadmium oxide NPs for 24 hr [84]. Similarly, a significant increase in pulmonary IL-6 was reported in rats exposed to silver NPs for 24 hr [86]. While cadmium oxide and silver NPs increased the pulmonary levels of these inflammatory cytokines, titanium dioxide is capable of inducing a significant increase in both the pulmonary and systemic levels of IL-6 and IFN-γ after 24–48 hr of exposure in a rat IT model [85]. Together, these reports demonstrate the capacity of pulmonary NP exposure to initiate systemic inflammation and highlight the potential influence that systemic inflammatory cascades may have on the outcomes of pulmonary NP exposure.

In contrast to the WC-Co NPs, CeO_2_ NPs induced significant acute pulmonary and systemic responses in our intra-tracheal instillation rat model. After 24-hr exposure, we observed significant acute inflammation in our CeO_2_ NP exposed group compared to the vehicle control in terms of LDH activity, albumin content, and macrophage activation state. These findings are consistent with a previous study in the Nurkiewicz laboratory [16], where significantly higher LDH, albumin, and number of activated AMs were observed after 24-hr exposure to 100–400 μg CeO_2_ NPs, which might have contributed to microvascular dysfunction [16]. The significant increases in AM activation and number of PMNs in this study indicated that CeO_2_ NPs stimulated the activation of macrophages and promoted the recruitment of PMNs. Furthermore, in this study, we found a significant increase in IL-6 levels in both the plasma and BAL fluid of CeO_2_ NP exposed animals compared to the vehicle control. This is most likely because IL-6 is primarily secreted by activated macrophages to stimulate inflammation in response to pulmonary tissue damage caused by the presence of NPs in the lung [61]. Overall, the outcomes reported here for CeO_2_ NPs are consistent with earlier studies regarding the systemic effects of exposure [16] and other *in vivo* [13, 51, 52] and *in vitro* [87–92] reports concerning CeO_2_ NP toxicity in the literature.

In the current *in vivo* study, WC-Co NPs did not induce significant acute pulmonary and systemic inflammation as originally hypothesized. We speculate that this could be due to a number of factors which were not examined fully in this preliminary study. In this case, we limited our investigation to a single IT dose (i.e. 50–500 μg per rat), representing an acute exposure to a total WC-Co NP lung burden which may accumulate in a person’s lungs after a period of exposure in an industrial environment. Further, our study focused on a short exposure time (i.e. 24 hr), so it remains possible that WC-Co NP exposure may cause a delayed response which was not observed at our 24 hr exposure time. In addition to delayed effects of WC-Co NP exposure, we speculate that the inflammatory state often observed in hard metal lung disease patients may be due to the chronic effects of WC-Co NP exposure due to NP accumulation in the lung over time, which may explain the overall lack of inflammation observed here after a single acute exposure to WC-Co NP for 24 hr. Future studies which explore the inflammatory effects of multiple WC-Co NP doses and/or longer exposure times are warranted to better define the pulmonary and systemic inflammatory response to WC-Co NPs *in vivo*.

## Conclusions

In this study, we examined the acute local pulmonary and systemic inflammatory responses to WC-Co NPs using an intra-tracheal instillation rat model. No significant differences between WC-Co exposed animals and vehicle control were observed in terms of LDH activity, albumin concentration, or cell differentials. Macrophages isolated from WC-Co animals also did not show significant activation when compared to macrophages from vehicle control animals. In addition, no significant differences in inflammatory cytokines were observed for WC-Co exposed animals. These findings indicated a lack of acute local pulmonary and systemic inflammatory responses after 24-hr exposure to WC-Co NPs in an IT dose in the range of 0–500 μg per rat.
